# Modulation of Membrane Microviscosity by Protein-Mediated Carotenoid Delivery as Revealed by Time-Resolved Fluorescence Anisotropy

**DOI:** 10.3390/membranes12100905

**Published:** 2022-09-20

**Authors:** Alexey N. Semenov, Danil A. Gvozdev, Dmitry V. Zlenko, Elena A. Protasova, Anastasia R. Khashimova, Evgenia Yu. Parshina, Adil A. Baizhumanov, Natalia Yu. Lotosh, Eleonora E. Kim, Yuriy N. Kononevich, Alexey A. Pakhomov, Alla A. Selishcheva, Nikolai N. Sluchanko, Evgeny A. Shirshin, Eugene G. Maksimov

**Affiliations:** 1 Faculty of Biology, M.V. Lomonosov Moscow State University, 1-12 Leninskie Gory St., Moscow 119991, Russia; 2 National Research Center “Kurchatov Institute”, 1 Acad. Kurchatov Sq., Moscow 123182, Russia; 3 A.N. Nesmeyanov Institute of Organoelement Compounds, Russian Academy of Sciences, Moscow 119991, Russia; 4 M.M. Shemyakin and Yu. A. Ovchinnikov Institute of Bioorganic Chemistry, Russian Academy of Sciences, Moscow 117997, Russia; 5 Federal Research Center of Biotechnology, Russian Academy of Sciences, 33 Leninsky Prospect, Moscow 119071, Russia; 6 Faculty of Physics, M.V. Lomonosov Moscow State University, 1-2 Leninskie Gory St., Moscow 119991, Russia; 7 Laboratory of Clinical Biophotonics, Biomedical Science and Technology Park, I.M. Sechenov First Moscow State Medical University, Trubetskaya Str. 8-2, Moscow 119991, Russia; 8 Institute of Spectroscopy, Russian Academy of Sciences, 5 Fizicheskaya Str., Troitsk, Moscow 108840, Russia

**Keywords:** carotenoproteins, carotenoid delivery, membrane microviscosity, time-resolved fluorescence anisotropy, FRET, molecular dynamics

## Abstract

Carotenoids are potent antioxidants with a wide range of biomedical applications. However, their delivery into human cells is challenging and relatively inefficient. While the use of natural water-soluble carotenoproteins capable to reversibly bind carotenoids and transfer them into membranes is promising, the quantitative estimation of the delivery remains unclear. In the present work, we studied echinenone (ECN) delivery by cyanobacterial carotenoprotein AnaCTDH (C-terminal domain homolog of the Orange Carotenoid Protein from *Anabaena*), into liposome membranes labelled with BODIPY fluorescent probe. We observed that addition of AnaCTDH-ECN to liposomes led to the significant changes in the fast-kinetic component of the fluorescence decay curve, pointing on the dipole-dipole interactions between the probe and ECN within the membrane. It may serve as an indirect evidence of ECN delivery into membrane. To study the delivery in detail, we carried out molecular dynamics modeling of the localization of ECN within the lipid bilayer and calculate its orientation factor. Next, we exploited FRET to assess concentration of ECN delivered by AnaCTDH. Finally, we used time-resolved fluorescence anisotropy to assess changes in microviscosity of liposomal membranes. Incorporation of liposomes with β-carotene increased membrane microviscosity while the effect of astaxanthin and its mono- and diester forms was less pronounced. At temperatures below 30 °C addition of AnaCTDH-ECN increased membrane microviscosity in a concentration-dependent manner, supporting the protein-mediated carotenoid delivery mechanism. Combining all data, we propose FRET-based analysis and assessment of membrane microviscosity as potent approaches to characterize the efficiency of carotenoids delivery into membranes.

## 1. Introduction

Carotenoids represent a wide class of pigments abundantly present in fungi, algae and both photosynthetic and non-photosynthetic plant cells [1]. They are also found in tissues and cells of insects, fish, crustaceans, mollusks, birds and are responsible for the coloration of these animals. Due to prominent anti-inflammatory and antioxidant properties, carotenoids are considered as promising therapeutic agents in the treatment of many severe pathologies such as cardiovascular [2] and neurodegenerative diseases [3].

Carotenoids are vital for the normal functioning of certain organs and tissues, serving as metabolic precursors of chemical compounds that determine various physiological processes. In particular, carotenoids form the basis for visual pigments responsible for light perception and color discrimination. In humans, carotenoids have been also detected in skin by non-invasive optical techniques [4,5] and in the yellow spot in the macular region of the retina [6]. Much attention has been attracted to the presence of various carotenoids in neural cells [7], including the human brain [8]. Earlier it was supposed that the main function of carotenoids in animal cells is quenching of reactive oxygen species (ROS), reactive nitrogen species (RNS), and other free radicals formed during oxidative stress. The major function of β-carotene, apart from acting as a lipid radical scavenger and singlet oxygen quencher, is being an optimal, naturally occurring provitamin A, which is essential for the normal tissue growth and development, immune function, and vision [9]. Xanthophylls, astaxanthin in particular, demonstrated a wide range of pharmacological activities in the human organism, including immunomodulatory and anti-cancer, hepatoprotective and cardioprotective effects as possible secondary manifestations of carotenoids’ antioxidant activity [10,11].

Carotenoids are synthesized by multienzyme complexes and function within photosynthetic reaction centers and light-harvesting antennas participating in the organization, functioning and regulation of photosynthetic apparatus [12], in particular, increasing the range of wavelengths accessible for light harvesting and assisting in photoprotection [13]. Mechanisms of carotenoid transfer from the area of their synthesis or absorption from dietary sources to places where they perform their functions are far from being completely understood.

Several different mechanisms outlined below reflect convergent evolution and dissimilarity of the carotenoid delivery systems. One mechanism, utilizing transfer of carotenoids (predominantly, astaxanthin) within lipid droplets that are transported from the inner part of the cell to the plasma membrane, was described for the microalgae *Haematococcus pluvialis* [14]. Its strains are widely used in modern biotechnology due to the profound ability to produce carotenoids under stress conditions [15]. However, there is still lack of knowledge on how these droplets are formed and transported from the central part of the cell to its periphery.

Another delivery mechanism utilizes water-soluble carotenoproteins capable of carotenoid transportation to specific cell compartments. Among such proteins from cyanobacteria is the photoactive Orange Carotenoid Protein (OCP) [16]. OCP is a 35 kDa protein consisting of two structural domains (N- and C-domain) connected by a long linker [17]. The single carotenoid chromophore is located in the inner space of the protein and spans both protein domains. Localization of the carotenoid occurs due to hydrogen bonds between the keto group of the carotenoid and the C-domains’ amino acids (tyrosine at position 201 and tryptophan—288, *Synechocystis* OCP numbering). Individual OCP domains preserve carotenoid binding and transportation activities in aqueous solutions, which led to the discovery of the protein-to-protein carotenoid transfer mechanism [18,19,20]. Recently, it was demonstrated that a natural homolog of the C-terminal domain of OCP from *Anabaena (Nostoc)* sp. PCC 7120 (hereinafter AnaCTDH protein) can uptake carotenoids (echinenone and canthaxanthin) from membranes of carotenoid-producing *E. coli* strains [21,22] and serve as an effective machinery for delivery of ketocarotenoids into cell membranes [23]. Exploitation of carotenoid-producing strains allowed to reconstitute holoforms of the ~20 kDa microalgal Astaxanthin-binding Protein (AstaP), which can accommodate several carotenoid types (astaxanthin, canthaxanthin, zeaxanthin, lutein and β-carotene) and transfer them to liposomes and apoforms of unrelated carotenoproteins from cyanobacteria [24]. In addition, it is known that the color of cocoons in silkworm *Bombyx mori* originates from carotenoids that are delivered by the intracellular ~30 kDa Carotenoid-Binding Protein (CBP) [25]. In recent work [26], functional CBP was reconstituted in *E. coli* and the capability of dose-dependent binding of lutein and zeaxanthin by this carotenoprotein was demonstrated in vitro. In addition, this protein can extract carotenoids from natural sources and deliver them to the apoform of OCP, making it photoactive, to the liposomes, or to the model fibroblast cells, improving their growth [27]. All these achievements clearly show that water-soluble carotenoproteins possess great potential to implement a high-effective platform for targeted delivery of various carotenoids into cell membrane for different applications.

Mechanisms of carotenoids absorption by human cells remain debatable. It may be driven by a concentration gradient, and passive diffusion in intestinal absorptive cells (enterocytes) is highly likely [28]. However, there are promising data on the existence of specific molecular protein-mediated mechanisms of carotenoids recognition, uptake, and transport in human cells. Recently, it was demonstrated [29,30,31], that in the Caco-2 cell line, a proxy for epithelial cells isolated from the colon tissue, such carotenoids as carotenes, lutein and astaxanthin can be transferred through the membrane via receptor-mediated mechanisms. In retinal cells, aster proteins (GRAM-domain-containing proteins), previously supposed to promote the transfer only of non-vesicular cholesterol between membranes, were shown to mediate carotenoid transport as well [32]. According to the modern data, β-carotene penetrates intestine epithelial cells through the scavenger receptor class B type 1 (SCAR-B1/SR-B1) transporter [4]. It was reported that human serum albumin is apparently capable of forming pigment-protein complexes with carotenoids [33,34] and the efficiency of the binding depends on the carotenoid type.

Since carotenoids are not synthesized in the human body, it is necessary to ensure their supply either as a part of the consumed products, or food additives and pharmacological substances. Efficient delivery of carotenoids into cells is limited due to the lipophilic nature of carotenoids, their poor solubility in water, low chemical/photochemical stability, and suboptimal bioavailability. Since membrane environment is natural for carotenoid molecules, which often act as modulators of physical properties of the membranes [35], the choice of the strategy of carotenoids selective delivery should consider not only the biophysical properties of the carotenoid, but also the peculiarities of the target membrane. For instance, the thickness of the membrane is a key factor determining the effects of polar carotenoids on mechanical and physical characteristics of the membrane [36]. The structure and the number of phospholipid components, which constitute the membrane, both determine the ratio between liquid-ordered and gel-ordered phases within the bilipid layer and, therefore, define the effects of carotenoids on membrane properties: e.g., dipolar carotenoids increase the order and decrease alkyl chain motion in fluid-phase membranes, but disorder lipids in gel-phase domains [37]. The presence of the cholesterol in membrane is also a very important factor since its presence tends to decrease the distance between phospholipids and thus affects the interaction between the membrane and carotenoid [38]. The saturation of fatty acids of membrane phospholipids is another feature which plays a role in the regulation of carotenoid delivery [39] because saturated lipids represent more ordered composition characterized with less distance between phospholipids. Overall, from this viewpoint, study of the mechanisms underlying the interaction between the various types of membrane and carotenoid molecules and carotenoids further intramembrane localization is essential for an adequate assessment of the targeted delivery of carotenoids into cells. Microscopic membrane viscosity (microviscosity) can be proposed as a relevant parameter, as it was found to be sensitive to membrane malfunctions observed on the cellular level at several pathologies, for example, during tumor malignization [40].

The aim of the present study was to develop a set of quantitative indicators of the efficiency of carotenoid delivery into membranes labelled with a fluorescent probe. We analyzed changes in parameters of the fluorescence of the probe (lifetime and intensity) caused by energy transfer between the carotenoid molecule and the fluorescent probe within the liposome membrane. As it should be considered that differences in the structural organization of different types of carotenoids may significantly affect not only the efficiency of penetration through the cell membrane, but also the structure and properties of the membranes themselves, we implemented molecular dynamics simulations for modeling of the behavior of carotenoid and fluorescent probe within the membrane, to study their localization and mutual orientation in the lipid bilayer. Next, we proposed an approach to estimate the number of carotenoid molecules transferred by carotenoprotein AnaCTDH into the liposome membrane, revealing the advantages of time-resolved fluorescence spectroscopy to estimate the efficiency of the selective delivery of carotenoids. To verify the delivery, we used time-resolved fluorescence anisotropy to investigate how different carotenoid types affect microviscosity of membrane of model liposomes. We also implemented alternative strategy of the delivery when carotenoid was directly loaded into liposomal membrane during liposomes synthesis, and measured microviscosity of such liposomes membranes to be able to additionally verify the protein-mediated carotenoid delivery. Combining all the obtained results, we propose that time-resolved fluorescence techniques provide novel opportunities to characterize the efficiency of the carotenoids target delivery into membranes.

## 2. Materials and Methods

### 2.1. Materials

**Figure 1:**
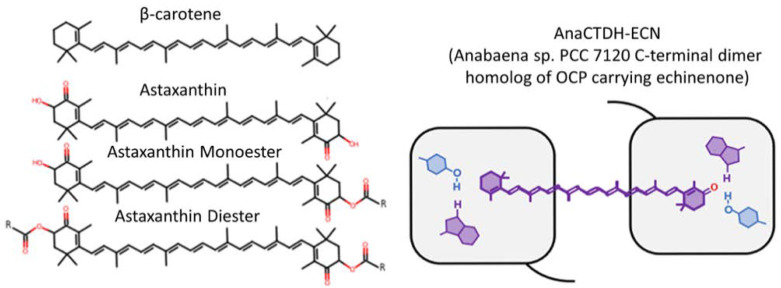
Structure of carotenoids and scheme of carotenoprotein AnaCTDH in holoform (loaded with echinenone) that were involved in the present study.

Structures of the studied carotenoids and the scheme of carotenoprotein AnaCTDH carrying echinenone are presented in Figure 1. Astaxanthin, its mono- and diester forms, and β-carotene were loaded directly into membrane during liposomes synthesis. Carotenoid powder of astaxanthin (SML 0982, Sigma Aldrich, Merck, Darmstadt, Germany); β-carotene (22040-5G-F, Sigma Aldrich, Merck, Darmstadt, Germany); separated mono- and diester forms of astaxanthin (separation protocol is described below) was weighted as 2 mg sample and dissolved in dichloromethane and subsequently, when incorporation of liposomes with carotenoid was required, added to the stock solution of phospholipid. Echinenone (ECN) was delivered into membrane via protein-mediated mechanism using carotenoprotein AnaCTDH. Holoforms of AnaCTDH were expressed in ECN-producing *E. coli* cells as described earlier [18]. Protein purification was performed by immobilized metal-affinity and size-exclusion chromatography to electrophoretic homogeneity and stored at 4 °C in phosphate buffered saline (pH 7.4) supplemented with 3 mM sodium azide as it was done previously [23]. Protein concentrations were determined by photometric method measuring absorbance at 280 nm, using calculated protein-specific molar extinction coefficient.

**Figure 2:**
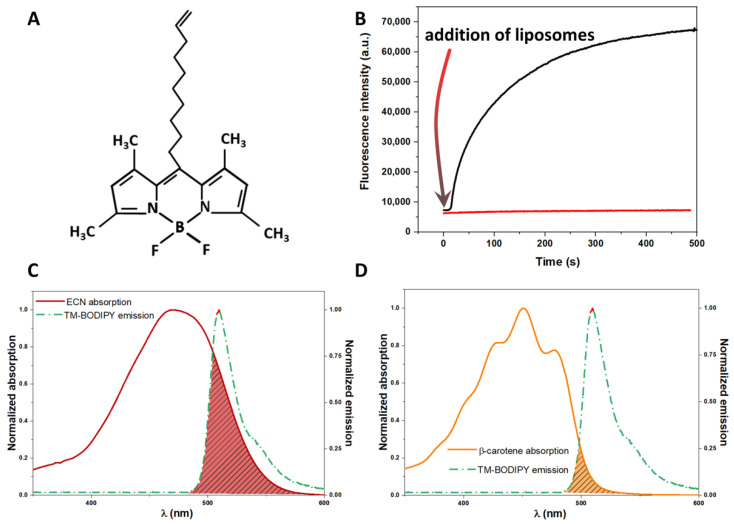
(**A**) Molecular structure of the *meso*-decene-1,3,5,7-tetramethyl-BODIPY (TM-BODIPY) probe that was used in the present study for membrane labelling; (**B**) Steady-state fluorescence of TM-BODIPY probe after addition of liposomes into aqueous solution; (**C**) The overlap of echinenone (ECN) absorption and TM-BODIPY emission spectra; (**D**) The overlap of β-carotene absorption and TM-BODIPY emission spectra.

*meso*-decene-1,3,5,7-tetramethyl-BODIPY, hereinafter TM-BODIPY (detailed description is provided in [41]), was used as a fluorescence probe to label liposomal membranes. It is a highly lipophilic molecule which fluorescence properties drastically depend on the local environment. Figure 2A shows the molecular structure of TM-BODIPY and Figure 2B demonstrates the time changes in the TM-BODIPY steady-state fluorescence at 1 μM concentration when liposomes were added into the solution of the probe. One can see that upon binding by the liposomal membrane, the intensity of the probe significantly increases, reaching saturation at approximately 10 min of incubation. The latter allows measuring properties of labelled membranes, considering the signal from the unbound probe to be negligibly small. Additionally, the emission spectrum of TM-BODIPY overlaps with echinenone (ECN, Figure 2C) and β-carotene (Figure 2D), suggesting that the mechanism of energy coupling as dipole-dipole interaction can be described by Förster theory of resonance energy transitions (FRET). The stock solution of TM-BODIPY was prepared by dissolving dry powder in 94% ethanol to achieve 6 mM concentration. To label liposomes, the stock solution of TM-BODIPY was diluted in advance with Milli-Q water to achieve the desired concentration of the probe in the experimental solution.

### 2.2. Preparation and Purification of Astaxanthin Mono- and Diesters

Extraction of mono- and diesters of astaxanthin was performed from mixture of astaxanthin mono- and diester forms (astaxanthin esters from *Haematococcus pluvialis*, 1044210, Sigma Aldrich, Merck, Darmstadt, Germany). The sample powder of the mixture was dissolved in hexane. The purification of astaxanthin fraction was performed using column chromatography on silica gel with particle sizes of 40–60 μm (Merck, Darmstadt, Germany) and gradient elution starting from 100% hexane to 80:20% hexane:acetone. The carotenoid type was determined by thin-layer chromatography and spectrophotometric techniques. The extraction of monoesters and diesters from the purified astaxanthin mixture was performed using high-performance liquid chromatography (HPLC, Agilent 1200 series, Agilent, Santa Clara, CA, USA) using detection at 470 nm.

Separation of astaxanthin monoester was performed using HPLC on the reverse-phase column Orbit-C18 (MZ-Analysentechnik GmbH, Mainz, Germany). Mobile phase represented 50:50% acetonitrile:water (Phase A1) and 100% acetonitrile (Phase B1). Elution rate was 700 μL/min, the volume of the injected sample was 20 μL. The elution program started at phase ratio 25:75% (A1:B1) and gradually continued during 12 min to isocratic elution with 100% B1. For more details, refer to [42]. Separation of astaxanthin diesters was performed on reverse-phase column Eclipse-C18 (Agilent, Santa Clara, CA, USA). Mobile phase represented 90:10% (Phase A2) and 98:2% (Phase B2) acetone:water. Elution rate was 800 μL/min, the volume of the injected sample was 20 μL. The elution was performed from 100% A2 to 100% B2 during 55 min according to the protocol described in [43].

### 2.3. Liposomes Synthesis Protocol

#### 2.3.1. Preparation of Nanoemulsion of Soy Lecithin Lipoid S75 Liposomes

Liposomes S75 were made from soy lecithin lipoid S75 (Lipoid GmbH, Ludwigshafen, Germany) representing a mixture of 75.3% phosphatidylcholine and 8% phosphatidylethanolamine. Control (intact) S75 liposomes without carotenoids were made from lipoid S75 powder dissolved in chloroform. To prepare S75 liposomes with carotenoids, a solution of phospholipid was supplemented with carotenoid/chloroform solution to achieve desired values (lipid concentration was 2 mg/mL, carotenoid concentration varied: 0.008–0.2 mg/mL for β-carotene; 0.002–0.25 mg/mL for astaxanthin; 0.25–0.8 mg/mL for astaxanthin esters). The solvent from the stock solutions was vaporized in a vacuum evaporator (Concentrator Plus, Eppendorf, Hamburg, Germany) to form a lipid film. Hot emulsification with ultrasound disintegration was performed to obtain liposomes from the lipid film. The film was supplemented with hot (70 °C) Milli-Q water and placed into ultrasonic bath Bandelin Sonorex (BANDELIN electronic GmbH & Co. KG, Berlin, Germany) for 15 min at 65 °C, with subsequent homogenization at 10,000 rpm for 1 min (Homogenizer, Daihan Scientific, Wonju, Korea). Next, the obtained raw nanoemulsion was put into an ultrasonic homogenizer (Sonics Vibra Cell, Sonics, Newtown, CT, USA) and disintegrated using the following settings of the device: pulse 1:1 (pulsing operating mode, pulsing duration (s): pulsing break time (s)); amplitude 30% (amplitude of the ultrasonic vibrations at the probe tip, percent from maximum); power 50 mJ. The ultrasound dispersing lasted 90 s and was repeated after a one-minute break. As a result, the suspension of liposomes was obtained.

#### 2.3.2. Preparation of Egg Yolk Lecithin Liposomes

Liposomes made of fresh raw egg yolk were used in the present work as an additional model system to supplement measurements performed on S75 liposomes to establish main general effects in experiments with much more heterogeneous systems. Egg lecithin liposomes were synthesized from fresh raw egg yolk lecithin using recommendations provided in [44] with minor modifications. A fresh raw egg was broken, and the inner content was carefully removed. Egg yolk was dissolved in 40 mL of cold (4 °C) acetone and homogenized for 10 min. The homogenate was stored in the refrigerator at 4 °C for 12 h. Next, the homogenate was centrifuged at 3000 rpm for 20 min at 4 °C. The precipitate was then resuspended in 40 mL of cold (4 °C) acetone and centrifugation repeated. Then the precipitate was placed in a thin layer on filter paper and the solvent was dried out for 2 h at room temperature. The dried powder was solved in 20 mL of a chloroform/methanol mixture (1:1) and carefully stirred for 30 min at room temperature. After stirring, the obtained solution was centrifuged at 3000 rpm for 20 min at 4 °C. The supernatant was separated and each 1 mL of supernatant was mixed with 3.75 mL of a 1:2 chloroform/methanol mixture and shaken thoroughly. Then 1.25 mL of chloroform was added and intense mixing repeated. Then 1.25 mL of distilled water was added and the obtained mixture was centrifuged at 1000 rpm for 5 min at 4 °C. After the centrifugation, the supernatant was discarded, leaving the organic bottom phase containing egg lecithin, which was then dissolved in chloroform to achieve a final concentration of approx. 20 mg/mL. 2 mL of this solution was put in a glass retort with a round bottom. The retort was set on rotor evaporator with water tank temperature at 30 °C and the chloroform was evaporated during 30 min. As a result, a thin phospholipid film was formed on the bottom of the retort. The film was solved in the buffer solution (50 mM Tris-HCl with 0.1 mM EDTA, pH 7.4). The obtained solution was then exposed to 7 cycles of freeze-thawing process. Each freeze-thaw cycle included freezing for 1.5 min at liquid nitrogen temperature (−196 °C) and subsequent thawing in thermostat at 37 °C until a complete defrost of the sample. Then 21 cycles of extrusion were performed using a 1 mL syringe for extrusion of the sample through the membrane with 1 μm-diameter pores until the sample becomes transparent and slightly opalescent.

To prepare egg yolk lecithin liposomes loaded with cholesterol, 2 mL of raw egg yolk lecithin, solved in chloroform at concentration 20 mg/mL, was mixed with 1 mL of cholesterol (C8667, Sigma Aldrich, Merck, Darmstadt, Germany), solved in chloroform at concentration 10 mg/mL. The obtained mixture was then put in a glass retort for vaporization in rotor vaporizer and afterwards the protocol of liposomes preparation was the same as described above.

#### 2.3.3. Assessment of Liposomes Size Distribution and Heterogeneity

Size distribution and homogeneity of produced liposomes were assessed by dynamic light scattering using Zetasizer Nano ZS (Malvern Instruments Ltd., Malvern, Great Britain).

### 2.4. Picosecond Time-Resolved Fluorescence Anisotropy Measurement

Membrane microviscosity was studied by assessing time-resolved anisotropy of TM-BODIPY probe fluorescence in liposome membranes with different phospholipid composition. Supplementary Figure S1 provides examples of the fluorescence anisotropy decay curves of TM-BODIPY in the aqueous solution of different types of liposomes. Anisotropy decay curves were measured to estimate the rotational diffusion correlation time θ of the probe within the liposomal membrane. This approach allows estimating microviscosity *η* of the lipid membrane [45,46,47] using the equation:

(1) $$\theta =\frac{4\pi \eta R^{3}}{3K_{B}T},$$

where θ—rotational correlation time, *K_B_*—Boltzmann constant, *T*—temperature, *η*—microviscosity of the membrane, *R*—size of the fluorescent probe.

**Figure 3:**
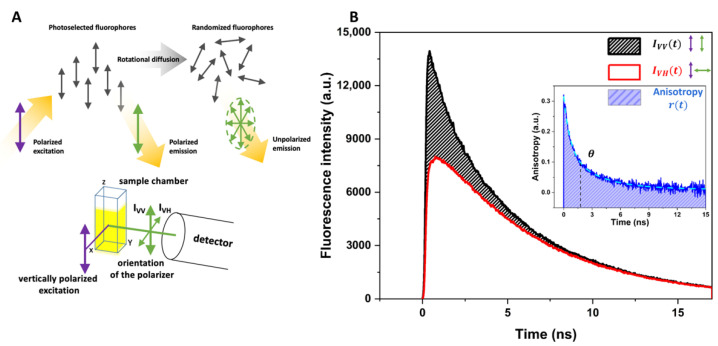
(**A**)—physical basis of the fluorescence anisotropy. When the sample is irradiated with polarized light (e.g., vertically), the emission light will be originated mostly from those fluorophores, which dipoles correspond to the direction of the excitation polarization and therefore constitute vertically polarized emission that is measured with vertical orientation of the polarizer positioned in front of the detector (designed as I_VV_). Due to the rotational diffusion of fluorophores and their mobility in the media, there will always be some components of unpolarized emission, which can be measured with orientation of the polarizer, different from vertical, for instance, at horizontal position assessing I_VH_. (**B**)—anisotropy kinetics (*r(t)*, blue graph) is formed by confronting decay curves of I_VV_(*t*) (black graph) and I_VH_(*t*) (red graph), and its relaxation time *θ* corresponds to rotational correlation time, which is proportional to the microviscosity. Cyan graph represents the approximation of anisotropy kinetics using double exponent decay function.

θ was obtained from the experimental data on anisotropy kinetics *r(t)* using approximation with a multiexponential decay function and corresponds to the mean relaxation time of anisotropy kinetics. Anisotropy kinetics *r(t)* was obtained by recording fluorescence decay curves corresponding to vertical and horizontal polarization of the detected fluorescence with picosecond time resolution [48]. When the set of fluorophores is irradiated with vertically polarized light, it will mostly excite those fluorophores, which dipoles are oriented closely to the polarization plane of the excitation light–photoselected fluorophores in Figure 3A. If fluorophores are absolutely ordered or immobilized, e.g., in crystals or in very viscous media, the emitted fluorescence will be correlated with excitation light and will be vertically polarized as well. However, if fluorophores can move freely or perform rotational movements, the degree of the emitted fluorescence polarization will be different from the excitation light and there will be components in the detected fluorescence signal corresponding to the non-vertical polarization fluorescence originated from randomized fluorophores. Measuring individually decay curves of vertically polarized fluorescence and non-vertical polarized fluorescence (in general, horizontally polarized, due to more simple and precise detection) and confronting them to each other (Figure 3B), one can obtain a new kinetics curve which is related to the correlation process between the ordered photoselected fluorophores and their randomized state:

(2) $$r\left(t\right)=\frac{I_{VV}\left(t\right)-GI_{VH}\left(t\right)}{I_{VV}\left(t\right)+2GI_{VH}\left(t\right)},$$

(3) $$G=\frac{I_{HV}}{I_{HH}},$$

where *G* is the factor of the detector sensitivity ratio towards vertical and horizontal light polarization. Total intensity was calculated as:

(4) $$I\left(t\right)=I_{VV}\left(t\right)+2GI_{VH}\left(t\right).$$

Kinetic curves were approximated using a multiexponential decay function:

(5) f(t)=∑iAie−tτi;r(t)=∑jA˜je−tθj,

where *f(t)* is fluorescence decay curve; r(t) is anisotropy kinetics; *A_i_* and ${\tilde{A}}_{j}$ are amplitudes of the corresponding components; $\tau_{i}$ and $\theta_{j}$ are characteristic (relaxation) decay times of *i*- and *j*-component, correspondingly. Average amplitude-weighted lifetime was used to characterize kinetics using the following equations:

(6) $$\tau_{av}=\frac{\sum_{i}A_{i}\tau_{i}}{\sum_{i}A_{i}};\theta =\frac{\sum_{j}{\tilde{A}}_{j}\theta_{j}}{\sum_{j}{\tilde{A}}_{j}}.$$

Fluorescence decay curves with picosecond time resolution were collected by time correlated single photon counting (TCSPC) setup comprised of single photon count board SPC-130EM paired with a hybrid detector HMP-100-07C with power supply controlled by DCC-100 module (all manufactured by Becker&Hickl, Berlin, Germany). Excitation at 500 nm was performed with a picosecond optical pulse generator (PLS-500-50, InTop, St. Petersburg, Russia) with 26 ps pulse duration driven at a repetition rate up to 25 MHz. A longpass filter with 550 nm cut-on wavelength (FEL0550, Thorlabs, Newton, NJ, USA) was used to block the excitation light. Detection was performed at 560 nm using monochromator ML-44 (Solar Laser Systems, Minsk, Belarus). The polarization of the excitation and emission light was performed by ultrabroadband wire-grid polarizers WP25M-UB (Thorlabs, Newton, NJ, USA), which position and rotation were supported by a motorized translator (K10CR1/M, Thorlabs, Newton, NJ, USA) and the rotational angle was controlled by specialized Thorlabs software (APT user, Thorlabs, Newton, NJ, USA).

Prior to the fluorescence anisotropy measurements, liposomes were labelled by TM-BODIPY probe. The labelling was performed by addition of 30 μL of the stock solution of liposomes into 1 mL of Milli-Q water containing 1 µM TM-BODIPY. After mixing, the final solution was incubated for 10 min at room temperature. Incubation and measurements of the samples were performed in a temperature-control cuvette holder Qpod 2e (Quantum Northwest, Liberty Lake, WA, USA).

AnaCTDH-ECN was added to the solution of already labelled liposomes. Concentration of AnaCTDH-ECN was varied by the stepwise addition of 1 μL aliquots of the stock solution. To establish carotenoid delivery, liposomes were incubated for 10 min after each step prior to fluorescence measurements.

### 2.5. Molecular Dynamics Modeling In Silico

In silico experiments involving molecular dynamics were conducted using GROMACS 2019.4 software package [49] in combination with CHARMM-36 force field [50]. Partial atomic charges in carotenoid and TM-BODIPY molecular models were estimated using RESP protocol [51]. The model egg yolk lecithin bilayer was assembled using CHARM-GUI engine [52] according to the approximate compositions available elsewhere [53]. The model lipid membrane composition is provided in Supplementary Table S1. The model membrane was dissolved in 50 mM NaCl solution (5 nm of the solution at each side of the bilayer).

After assembling the model, the first relaxation simulation was conducted (10 ns, NVT, all atoms heavier than hydrogen of the bilayer fixed) for the solvent relaxation. Then, the second relaxation simulation (10 ns, NPT) was conducted using stable but not accurate pressure coupling procedure in accordance with the Berendsen algorithm. After the relaxation, a production simulation period of at least 1 µs was conducted using correct pressure and temperature protocols. The simulation step was 2 fs, and the corresponding restraints were applied to the H-bonds (using recommendations from [54]). The effective orientation factors for pairs of the molecules were calculated directly as the time-averaged means of the corresponding conformations, observed during the whole stimulation.

### 2.6. Spectroscopy Measurements

Absorption and emission spectra were obtained using a Flame UV-VIS spectrometer (Ocean Insight, Orlando, FL, USA). Absorption spectra measurements involved illumination using a fiber-coupled white light source SLS201L/M (Thorlabs, Newton, NJ, USA).

### 2.7. Data Analysis

Fluorescence decay curves were analyzed using the SPCImage (Becker&Hickl, Berlin, Germany) software and OriginPro 2021 (OriginLab Corporation, Northampton, MA, USA). Analysis of the spectroscopic data was performed in SpectraSuite (Ocean Insight, Orlando, FL, USA) software. All measurements for each type of liposomes and carotenoid were performed in triplicates, error bars included standard deviation, instrumentation error and error of approximation. Chemical structures of carotenoids were visualized using rdkit.Chem.PyMol module and Inkscape 1.0.2. The numerous analyses of distances between donor/acceptors within FRET, including calculations of Förster distance, were performed in PhotochemCAD software.

## 3. Results

### 3.1. Delivery of Carotenoid Molecules in the Liposomal Membrane Studied by FRET

**Figure 4:**
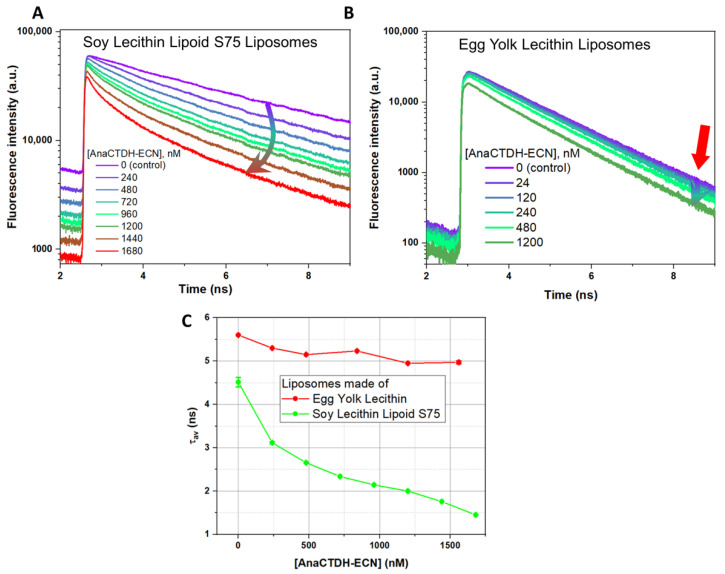
HeteroFRET induced effects between echinenone (ECN, acceptor), delivered by carotenoprotein AnaCTDH into membrane of liposomes, and the fluorescent probe TM-BODIPY (donor, concentration 1 µM) at different concentrations of AnaCTDH-ECN: fluorescence decay curves of TM-BODIPY in (**A**) soy lecithin lipoid S75 liposomes and (**B**) in egg yolk lecithin liposomes (presented in the semi-log scale); (**C**) comparative changes in TM-BODIPY fluorescence average lifetime values τ_av_. All measurements were performed at 20 °C.

In our system of liposomes with the fluorescence probe, the addition of carotenoid changed TM-BODIPY fluorescence lifetime, which indicates possible excitation energy transfer from TM-BODIPY molecule to the carotenoid (Figure 4). Figure 4A,B demonstrate fluorescence decay curves of TM-BODIPY in S75 liposomes and egg yolk lecithin liposomes, correspondingly, after incubation at different concentrations of AnaCTDH-ECN. Fluorescence decay curves in control solution without carotenoids were mono-exponential, while in the presence of AnaCTDH-ECN two kinetic components manifested. τ_av_ decreased in both types of liposomes (Figure 4C). In egg yolk lecithin liposomes without carotenoid (control), τ_av_ = 5.601 ± 0.007 ns, and it decreased to 4.971 ± 0.035 ns when liposomes were incubated with 1560 nM AnaCTDH-ECN. In S75 liposomes, τ_av_ decreased from 4.51 ± 0.11 ns (control, without carotenoids) to 1.450 ± 0.04 ns at 1680 nM AnaCTDH-ECN. According to the patterns of fluorescence decay curves, it is clearly seen that the decrease in τ_av_ was caused by changes in the fast-kinetic component τ_1_, which is related to the energy transfer from TM-BODIPY (donor) to ECN (acceptor), while the long component τ_2_ did not change significantly.

### 3.2. Molecular Dynamics Simulations of Localization and Distribution of Carotenoids within the Hydrophobic Core of the Model Membrane

The decrease in the fluorescence lifetime, observed in fluorescence decay curves at different concentrations of AnaCTDH-ECN (Figure 4), clearly indicates a reduction in the average distance between the fluorescence probe TM-BODIPY and the carotenoid as the concentration of AnaCTDH-ECN increases in the solution of liposomes. To quantify observed effects of energetic coupling between TM-BODIPY and carotenoids, we estimated their distribution and localization in liposomal membrane using molecular dynamics to assess the orientation factors of the chromophores within the hydrophobic core of the membrane.

**Figure 5:**
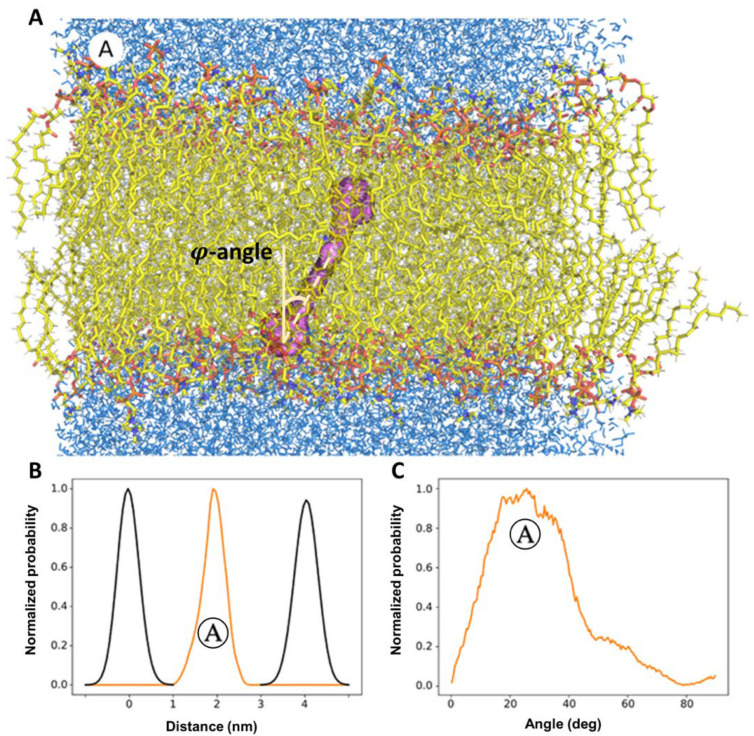
Results of molecular dynamics simulations of equilibrium localization of echinenone (ECN) molecule in the bilipid layer. Data was visualized based on the analysis of the trajectory of the center-mass of ECN molecule. (**A**)—possible localization of ECN in the membrane where it occupies only one position (designed as state Ⓐ) during the whole exposition of the stimulation. (**B**)—the probability density function (orange-colored curve) for the localization of the ECN center of mass along the normal to the membrane plane, black-colored curves represent the boarders of the bilipid membrane. (**C**)—the probability density function for the *φ* angle between the long axis of the molecule and the normal to the membrane plane.

Figure 5 demonstrates the results of molecular dynamics simulations of the motion of ECN molecule in the model bilipid membrane. Histograms of probability densities were obtained by analyzing the trajectory of the center mass of the carotenoid molecule. ECN occupies only one position in the bilipid membrane (Figure 5A,B) during the computational time period (1 μs). This position corresponds to state Ⓐ which is characterized with wide distribution of φ (orientation angle between the axis of the chromophore polyene chain and the normal laying in the membrane plane) with peak position at ca. 30° (Figure 5C). ECN is a polar carotenoid and less hydrophobic, which can explain the predominance of such localization in the membrane. A very similar localization was reported in the recent work by Grudzinski et al. [55] for zeaxanthin. Next, the propagation of TM-BODIPY molecule inside the lipid bilayer, loaded with ECN, was modelled. The results are presented in the Supplementary Video. It is clearly seen, that TM-BODIPY propagates in the hydrophobic core of the membrane almost freely and can be located very closely to the ECN molecule, supporting a FRET-based mechanism.

**Figure 6:**
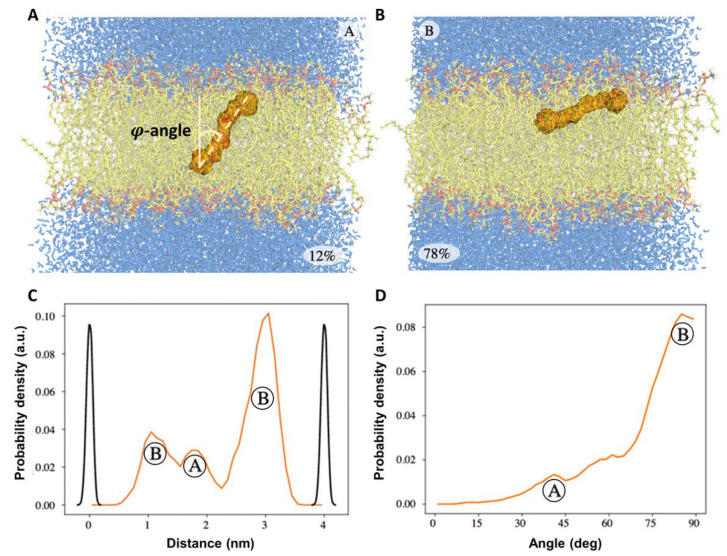
Results of the molecular dynamics simulations of equilibrium localization of β-carotene molecule in bilipid layer. Data was visualized based on the analysis of the trajectory of the center-mass of β-carotene molecule. (**A**,**B**)—possible localizations of β-carotene molecule in the membrane where it occupies either state Ⓐ (**A**) or state Ⓑ (**B**) position. State Ⓑ is more probable, as it was realized for approximately 78% of the simulation time. (**C**)—the probability density function (orange-colored curve) for the molecule center of mass localization along the normal to the membrane plane, black-colored curves represent the boarders of the bilipid membrane. (**D**)—the probability density function for the angle *φ* between the long axis of the molecule and the normal to the membrane plane.

Figure 6 demonstrates the results of molecular dynamics simulations of the motion of a β-carotene molecule in the model phospholipid membrane. We can see that localization of β-carotene in the membrane is completely different in comparison with ECN. One can observe that there are two possible localizations of β-carotene in the bilipid layer, corresponding to state Ⓐ (Figure 6A) and state Ⓑ (Figure 6B). State Ⓑ was found to be more probable because, according to the probability density diagram of localization (Figure 6C), it was observed among two variants of repositioning as the most probable positioning being realized at approximately 78% of the simulation time (right peak on the diagram) and thus is supposed to be more energy efficient. State Ⓐ corresponds to the ca. φ = 40°–45°, while state Ⓑ corresponds to nearly φ = 80°–85° (Figure 6D). The obtained results on this bimodal distribution of β-carotene molecule in the bilipid layer correspond with the experimental data: it was shown by angle-resolved resonance Raman spectroscopy that β-carotene, depending on the phospholipid composition of the membrane, can be oriented either parallel to the bilayer plane (e.g., in dioleoyl lecithin membrane) or perpendicular to it (in soybean lecithin) [56].

### 3.3. Targeted Delivery of Carotenoids via Carotenoprotein: Estimation of Efficiency

We attempted to estimate the carotenoid concentration in liposomes considering excitation energy transfer between TM-BODIPY and ECN and the data on carotenoids orientation in the membrane obtained from molecular dynamics simulations. To achieve this, we used the fact that there is an overlap between carotenoid absorption and TM-BODIPY emission spectra (Figure 2C for ECN and Figure 2D for β-carotene), and probability of FRET between donor (fluorescent probe) and acceptor (carotenoid); suggesting TM-BODIPY in the absence of aggregation has the quantum yield = 0.95 (taken from [57,58]); assuming molar extinction coefficient of β-carotene being equal to 125,300 M^−1^·cm^−1^ and ECN to 75,300 M^−1^·cm^−1^ (data was taken from [59]); and setting the refractive index to 1.4251 for the soy lecithin emulsion environment (based on the data from [60]). Next, the orientation factor χ^2^ between molecular transition dipole moments is usually used as 2/3 in randomly oriented systems, but in our case, there may be preferred orientations of carotenoid and fluorescence probe in the membrane. The orientation of such long, bar-shaped molecules depends very much on the extent of the substitution on the polar end-group, and the ability to form hydrogen bonds within the polar headgroup zones of the membrane. It is commonly accepted that there is no general rule for the orientation of highly hydrophobic non-polar β-carotene within a lipid bilayer [61]. One of the most probable variants is location mostly parallel to the membrane surface in the center of the phospholipid bilayer between the hydrophobic tails of phospholipid molecules [62]. Our molecular dynamics results support such representation of β-carotene localization (Figure 6). Molecular dynamics computational tests performed for ECN localization (Figure 5) described sloped positioning within the hydrophobic core at an orientation angle in the range ca. 25°–33°. The mechanism underlying such localization can be referred to a hydrophobic mismatch between the membrane thickness and the distance between the terminal hydroxyl groups of echinenone. Another possible explanation can be related to the presence of oxygen in the head groups. Using the overall results of molecular dynamics modeling, we were able to establish the average (according to all possible conformations of the molecule, observed during the stimulation process) orientation factor χ^2^ which was calculated for β-carotene and echinenone and was equal to 0.49 ± 0.17 and 0.68 ± 0.28, correspondingly. Thus, calculated values of Förster distances (a distance between donor and acceptor molecules at which FRET efficiency is 50%) between TM-BODIPY and carotenoid were $R_{0}^{ECN}=59\;Å$ for echinenone and $R_{0}^{\beta -car}=44\;Å$ for β-carotene.

The real FRET efficiency (W) can be calculated from changes in fluorescence lifetime *τ* of the TM-BODIPY fluorescence upon carotenoid addition to the liposome (Figure 4C) using formula:

(7) W=1−τD+AτD, 

where $\tau^{D}$—the average fluorescence lifetime of TM-BODIPY (donor) without carotenoid; $\tau^{D+A}$—the average fluorescence lifetime of TM-BODIPY with carotenoid (acceptor) addition.

**Figure 7:**
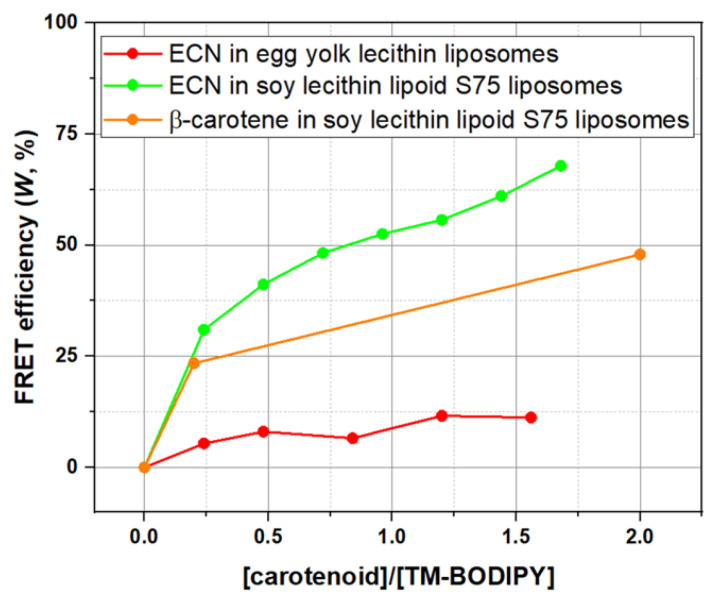
Dependence of FRET efficiency between carotenoids (echinenone (ECN) or β-carotene) and TM-BODIPY on the molar concentration ratio between them, measured in liposomes with different phospholipid composition.

From the experimental data on lifetime values of TM-BODIPY fluorescence in soy lecithin liposomes S75 for 0.2 molar ratio between carotenoid and fluorescence probe (molar ratio calculated from initial values of TM-BODIPY and carotenoids in the solution), FRET efficiency (*W*) was found to be equal to 23.4% for β-carotene and 31.0% for ECN (Figure 7). Here, it is important to mention, that, in contrast to ECN, β-carotene was loaded into liposomes directly during the liposome synthesis. Now, we can estimate the average distance (R) between the carotenoid and the TM-BODIPY molecule within the membrane of soy lecithin liposome:

(8) $$R=\sqrt[{6}]{\left(\frac{R_{0}^{6}}{W}-R_{0}^{6}\right).}$$

**Figure 8:**
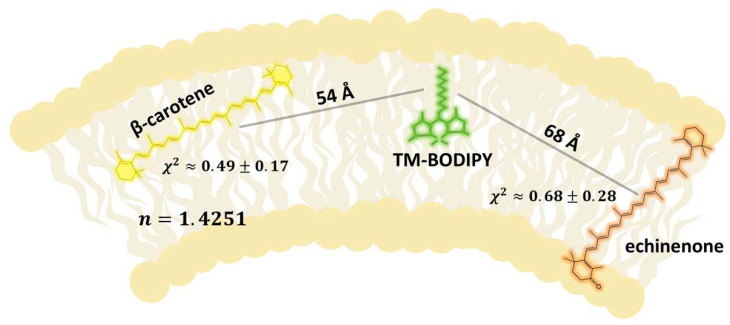
Graphical illustration of the localization of TM-BODIPY, β-carotene and echinenone in the phospholipid membrane. Indicated average orientation factor ($\chi^{2}$) corresponds to the mutual position of carotenoid and fluorescent probe in the membrane according to molecular dynamics simulations. Average distances between molecules were calculated based on FRET efficiency assessed in the experiment and calculated value of Förster distances. *n* is a refractive index which corresponds to soy lecithin emulsion environment.

Calculations using Formula (8) give us ECN average distance $R^{ECN}=68\;Å$ and for β-carotene $R^{\beta -car}=54\;Å$. Combining all the estimations, we can describe the localization of carotenoids in relation to TM-BODIPY probe within liposomal membrane (graphical illustration is provided in Figure 8).

In addition, usage of FRET allows us to estimate the minimum number of carotenoid molecules delivered into liposomes membrane necessary to ensure quenching of the TM-BODIPY fluorescence with a given efficiency. As it is seen from fluorescence decay curves (Figure 4A,B), in the presence of AnaCTDH-ECN we observe the appearance of the fast component originated from TM-BODIPY molecules, participating in the energy transfer. It means that the amplitude $A_{1}$ of the fast component corresponds to the share of TM-BODIPY molecules taking part in the energy transfer. At this point, we assume that all molecules of TM-BODIPY were incorporated in the membrane (such situation is realized during the incubation of liposomes with TM-BODIPY when the total fluorescence intensity measured in the labelled liposomes sample reaches maximum). Analysis of the fluorescence decay curves shows that for molar ratio [AnaCTDH-ECN]:[TM-BODIPY] = 0.2 the value of amplitude $A_{1}$ = 18.19%. Then the concentration of FRET-participating TM-BODIPY will be 182 nM. This concentration is calculated to the whole volume of the aqueous solution in the probe, meaning that local concentration of the chromophore might be higher. In the assumption that the energy transfers from 1 donor to 1 acceptor, the minimal concentration of ECN molecules participating in FRET will also be 182 nM.

### 3.4. Effects of Different Carotenoids on Microviscosity of Liposome Membrane Revealed by Fluorescence Anisotropy

**Figure 9:**
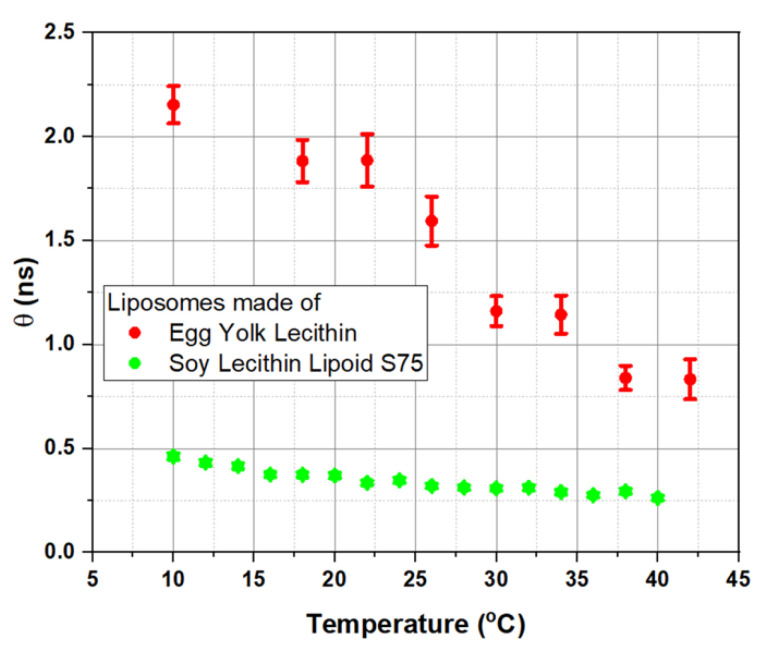
Changes in microviscosity of membranes of liposomes with different phospholipid compositions revealed by time-resolved fluorescence anisotropy of TM-BODIPY probe at 1 µM concentration. *θ* is rotational correlation time. Measurements were performed on two types of liposomes: green marks—S75 liposomes, produced from soy lecithin lipoid S75 containing 75.3% phosphatidylcholine and 8% phosphatidylethanolamine; red marks—liposomes prepared from fresh egg yolk lecithin (for the data on the composition please refer to [64]).

Figure 9 demonstrates the temperature dependence of the rotation correlation time *θ*, measured for soy lecithin S75 liposomes (S75) and egg yolk lecithin liposomes. The temperature effect was more pronounced for egg yolk liposomes: at every temperature, *θ* values of egg yolk liposomes were higher than in S75 liposomes. It indicates that microviscosity of egg yolk liposomes was higher than microviscosity of membranes of soy lecithin-made S75 liposomes, as the mobility of TM-BODIPY probe was significantly limited. This result cannot be related directly to the type of lecithin, as in general it does not influence the viscosity [63]. In addition, TM-BODIPY molecule propagates inside the lipid bilayer of nano-sized liposomes almost freely (according to molecular dynamic simulations visualized in Supplementary Video) and at concentrations lower than 1 μM it is not prone to aggregation and heterooligomerization, which could have affected its fluorescence intensity and lifetime [58]. HomoFRET can affect *θ* values (Supplementary Figure S2), but still it should not significantly alter experimental results on microviscosity since all the measurements were performed under the same conditions. So, we can conclude that observed changes in correlation time between two different types of liposomes are caused by the presence of various additional minor components among the major constituents of the membrane. To test this assumption and further validate the method, we performed measurements of membrane microviscosity of egg yolk liposomes loaded with cholesterol. The results are presented in Supplementary Figure S3. Indeed, the addition of cholesterol into liposomal membrane led to a significant increase in microviscosity. Moreover, the addition of cholesterol provided a phase transition at 30 °C. It supports the idea that the presence of additional constituents such as cholesterol and\or carotenoids in the bilipid layer of model liposomes may drastically modulate its properties which is characterized with increased *θ*.

**Figure 10:**
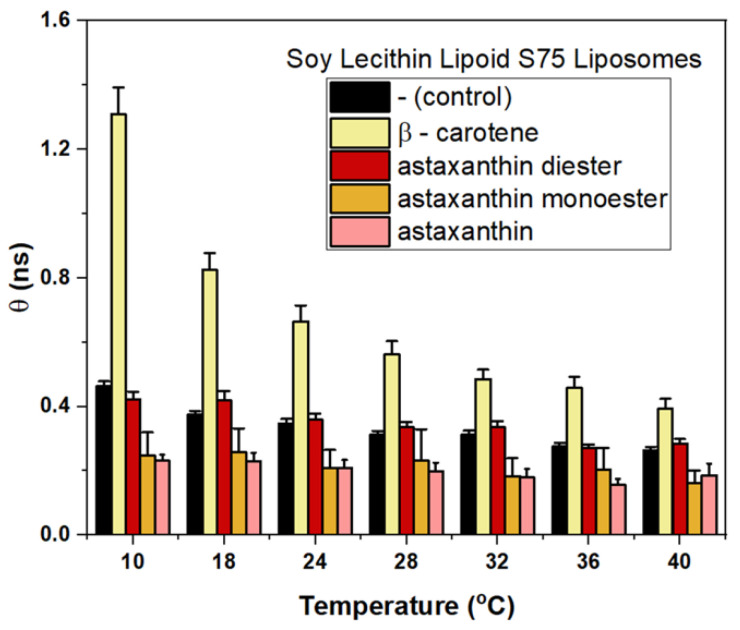
Effects of loading S75 liposomes with different carotenoids on membrane microviscosity as revealed by time-resolved fluorescence anisotropy of TM-BODIPY probe at 1 µM concentration. *θ* is rotational correlation time. Carotenoids were loaded into liposomes during their synthesis. Control values were obtained on intact liposomes without carotenoids.

To estimate the effect of different carotenoids, *θ* was measured for S75 liposomes preloaded with β-carotene, astaxanthin, astaxanthin monoester and astaxanthin diester (Figure 10) at the stage of liposome production. This step was required to verify *θ* as an adequate parameter, sensitive to the presence of carotenoids within the lipid bilayer. In each case, the amount of lipid and carotenoid were adjusted to achieve equal lipid:carotenoid concentration ratio of 2.0:0.2 mg/mL. Measurements of *θ* were performed in the temperature range from 10 to 40 °C. As it can be seen, the effect on *θ*, and, correspondingly, on the liposome membrane microviscosity, depends on the type of the carotenoid loaded into the membrane. Among all studied carotenoids, only β-carotene had a significant effect demonstrating an increase in *θ*. It means that rotational diffusion of the TM-BODIPY probe immersed in the lipid membrane core, represented by an increase in the correlation time, is a demonstration of the restriction to a molecular motion of lipids that can be caused by the presence of β-carotene, which corresponds with the data available in the literature [61].

**Figure 11:**
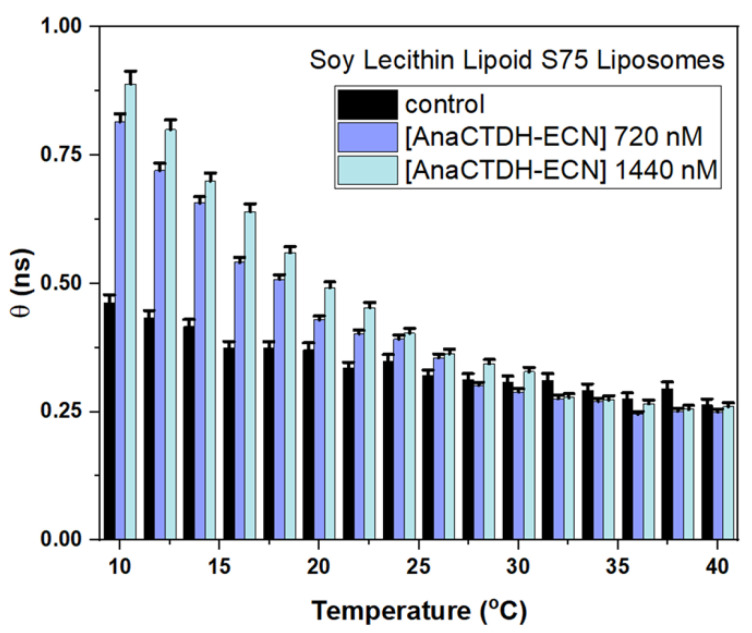
Effect of protein-mediated delivery of carotenoid (echinenone, ECN) using carotenoprotein AnaCTDH on the membrane microviscosity of soy lecithin S75 liposomes revealed by time-resolved fluorescence anisotropy of TM-BODIPY probe at 1 μM concentration. *θ* is rotational correlation time. Control values were obtained on intact liposomes without carotenoids.

Next, the effects of the protein-mediated delivery of carotenoid (echinenone) on microviscosity of liposome membrane were evaluated using time-resolved fluorescence anisotropy. Results are presented in Figure 11. It is seen that incubation of intact liposomes S75 (control) with AnaCTDH loaded with ECN (AnaCTDH-ECN) increased membrane microviscosity in a dose-dependent manner. The effect was clearly detected at low temperatures; however, it decreased at high temperatures, and in the physiological range almost disappeared.

## 4. Discussion

Since the localization of carotenoids in the bilipid membrane determines their protective and antioxidant effects [65], knowledge about the mechanisms of their distribution in the membrane is important. In the present study, we incorporated carotenoid molecules into two different types of liposomes by utilizing different approaches: direct enrichment of the membranes upon the production of liposomes in the presence of specific carotenoids (β-carotene, astaxanthin and its mono- and diester forms) or via the protein-mediated delivery module AnaCTDH carrying echinenone (AnaCTDH-ECN).

When the carotenoid was directly incorporated into the membrane of liposomes during their production, the phospholipid system became less homogeneous in terms of PDI (polydispersity index), and the size of the lipid particles increased. Based on the obtained results (Supplementary Table S2), it can be concluded that the homogeneity and mean size of the nanoemulsion depend on the ratio between lipid and carotenoid.

The direct incorporation of β-carotene into liposomes increased membrane microviscosity over the temperature range 10–40 °C. Such an effect is generally accepted as common for non-polar carotenoids, like β-carotene or lycopene, due to their specific localization in the membrane [65]. Carotenoids positioned horizontally in the membrane (which is realized for β-carotene as it is seen in Figure 6B) simultaneously interact with numerous hydrophobic fatty acid tails, leading to limited diffusion of the carotenoids themselves and other hydrophobic molecules within the membrane. Such an effect is clearly pronounced in membranes with the thickness of the hydrophobic core comparable with the length of the carotenoid molecule or lower. Additionally, this effect can be enhanced by the ability of carotenoids to form spatial aggregate-like structures affecting rotational diffusion [66]. Positioning of non-polar carotenoids in the membrane is very similar to the localization of cholesterol [35], and therefore, the effect of β-carotene was found to be comparable to the effect of cholesterol at temperature below the phase transition (see Supplementary Figure S3, where loading of the liposomes with cholesterol is shown to significantly increase membrane microviscosity).

Compared to β-carotene, astaxanthin is less hydrophobic due to the presence of hydroxyl and carbonyl groups in both ionone rings. In the temperature range from 10 to 40 °C, the inclusion of astaxanthin or its mono- and diester forms in the liposomal membrane did not increase *θ*. In numerous studies [35,55], it is assumed that astaxanthin is located in the phospholipid membrane almost perpendicular to its plane, so that the ionone rings are located in the polar layer of the membrane. Such localization is supported by the polarity and length of the astaxanthin molecule, and by the dependence of the carotenoid orientation angle on the thickness of the hydrophobic core of the liposomal membrane. It is possible, that astaxanthin is able to interact with the membrane upper surface layer, which is enriched with water molecules. For astaxanthin mono- or diester forms we can speculate that the angle *φ* between the axis of carotenoid molecule and the normal to the membrane plane (orientation angle) would be even more sharp to support the transmembrane orientation. The orientation angle negatively correlates with the thickness of the hydrophobic core of the membrane [67]: the greater the thickness of the membrane, the lower the orientation angle. Such correlation can be interpreted as a demonstration of the general rule that the orientation of polar carotenoids is determined by a matching of the distance between the opposite polar groups of the carotenoid and the thickness of the hydrophobic core of the membrane [67]. The astaxanthin diester has two additional (comparing to astaxanthin) fatty acid moieties, and its action practically coincides with the control. This fact can be explained by the mechanism proposed in [55] for lutein, a polar carotenoid which exhibits a higher affinity to be localized in the intermembrane region. On the other hand, such structural additives in diester form of astaxanthin should not completely deprive oxygen atoms from their partial negative charge, which should additionally prevent the horizontal localization of the carotenoid molecule in the hydrophobic core of the membrane. Further studies of the behavior of polar carotenoids in complicated, heterogeneous environments are warranted.

Time-resolved fluorescence anisotropy revealed that incubation of liposomes made from soy lecithin lipoid S75 with the AnaCTDH carotenoprotein carrying echinenone changed the liposomal membrane microviscosity by increasing the rotation correlation time. This serves as evidence for the effective echinenone delivery by AnaCTDH. The effect of AnaCTDH-ECN on membrane microviscosity at temperatures >30 °C was found to be insignificant. It cannot be related neither to the temperature degradation of AnaCTDH at such temperatures nor to the inhibition of the delivery process, as it was proved using optical spectroscopy that AnaCTDH is capable to deliver ECN to liposomal membrane at temperatures up to 40 °C [23]. Such a result can be explained that at low temperatures, separation of phospholipids into different phases occurs in a mixture of natural phospholipids which constitute lipoid S75 used in the present work. For a binary phospholipid mixture, such phases can be referred to as gel-ordered and liquid-ordered domains [68]. Molecules upon interaction with the membrane have different partitioning preferences between lipid phases, depending on the specific lipid host system [69]. In particular, such behavior was observed for BODIPY-containing probes, which suggests different ability to detect specific lipid phases [70]. Therefore, we can suggest that at low temperatures, TM-BODIPY and echinenone molecules were localized in one lipid phase, and thus changes in membrane viscosity are most efficiently detected. At high temperatures, lipid phases differentiation disappeared, and collocation between TM-BODIPY and echinenone changes, and the ability to detect changes in viscosity, originated from echinenone, decreases. Thus, the fact of successful carotenoid delivery into liposomes by AnaCTDH carotenoprotein can be established by observing a clear effect on the time-resolved fluorescence parameters of TM-BODIPY at temperatures up to 30 °C. However, the mechanisms of the temperature-driven changes in membrane microviscosity at different concentrations of AnaCTDH at higher temperatures should be further investigated on a model membrane objects with the homogenous phospholipid composition.

Since the addition of carotenoids at the stage of liposome synthesis makes it impossible to accurately assess the concentration of embedded carotenoids due to their low stability and yield of the resulting liposomes, protein-mediated delivery opens up a number of new additional experimental modalities. The mobility of TM-BODIPY allows its localization around ECN at the distance close enough to implement FRET (Supplementary Video). Therefore, FRET provides indicative information on the efficiency of the carotenoid delivery. However, the choice of the fluorescent probe concentration should be carefully adjusted to avoid changes in fluorescence intensity, lifetime, and anisotropy induced by homoFRET. Supplementary Figure S2 provides the data on total intensity, mean lifetime and rotation correlation time of TM-BODIPY in S75 and egg yolk lecithin liposomes, depending on concentration of the probe. We can propose significant alterations of fluorescence parameters at TM-BODIPY concentration above 1000 nM. The mechanism underlying this effect is mostly caused by homoFRET interactions between TM-BODIPY molecules within the liposome membrane when they are located in the membrane closer than 5 nm from each other, which is achieved at high concentration of the probe. Additionally, possible oligomerization or bulk aggregation of TM-BODIPY monomers can also be the case. All of these factors should be considered while developing the protocol of liposomes labelling to achieve a balance between stable labelling and sufficient intensity values and, if possible, avoid significant alterations of measured fluorescence. Noticeably, the magnitude of these side effects also depends on the viscosity of liposomes: they were more pronounced in less viscous S75 liposomes in comparison with more rigid raw egg yolk lecithin liposomes.

## 5. Conclusions

Nowadays, various methods are proposed to deliver carotenoids into the cytoplasmic membrane of the cells [71,72,73,74,75]. Yet, there is a lack of quantitative methods to evaluate the efficiency of the carotenoid delivery into the membrane. In the present work, we demonstrated that liposomal membrane microviscosity measured by time-resolved fluorescence anisotropy was sensitive to the presence of various carotenoids, including those that were delivered by protein-mediated mechanisms implemented in the aqueous media. We used this approach combined with molecular dynamics and FRET data analysis to estimate the efficiency of protein-mediated carotenoid delivery. We demonstrated a high efficiency of carotenoid delivery via protein-mediated mechanisms, clearly indicating a significant potential of using carotenoproteins as selective-delivery modules in different biomedical applications. The observation of the FRET-induced changes in the parameters of the fluorescence lifetime originated from the interaction between the fluorescent probe and carotenoid within the membrane provides new opportunities to study localization and concentration of carotenoid in the hydrophobic core of the membrane upon its uptake and thus allows us to estimate the efficiency of the target delivery of carotenoids.

## Data Availability

Not applicable.

## Supplementary Materials

The following supporting information can be downloaded at: https://www.mdpi.com/article/10.3390/membranes12100905/s1, Figure S1: Anisotropy curves of TM-BODIPY fluorescence probe (concentration 1 µM) docked in the membrane of soy lecithin lipoid S75 liposomes: (A)—loaded with various carotenoids during the synthesis (lipid:carotenoid concentration 2.0:0.2 mg/mL); (B)—incubated with carotenoprotein AnaCTDH-ECN. Measured at 20 °C. (C)—anisotropy curves of TM-BODIPY in egg yolk lecithin liposomes, incubated with AnaCTDH-ECN. Measured at 20 °C. (D)—rotational correlation time θ of TM-BODIPY probe in different liposomes depending on concentration of AnaCTDH-ECN. Measured at 20 °C. Figure S2: Dependence of total intensity, mean lifetime and correlation rotation time on concentration of TM-BODIPY probe in different liposomes (role of homoFRET effects); Figure S3: Effect of cholesterol, loaded into raw egg yolk liposomes, on membrane microviscosity (A) and occurrence of phase transition (B). Table S1: Parameters of the phospholipid membrane that was used in molecular dynamics computational stimulations; Table S2: Size distribution and polydisperse index (PDI) of studied liposomes. Video S1: Visualization of the molecular dynamic simulations of localization of echinenone and TM-BODIPY molecules within the hydrophobic core of the bilipid membrane and their interaction with each other.
