# Supplementary material for: Modulation of Membrane Microviscosity by Protein-Mediated Carotenoid Delivery as Revealed by Time-Resolved Fluorescence Anisotropy
Source: Membranes (Basel). 2022 Sep 20;12(10):905. doi: 10.3390/membranes12100905 (PMC9609150; doi:10.3390/membranes12100905)
Supplement: Supplementary file 1 [file membranes-12-00905-s001.zip › Supplementary Information_final.pdf]

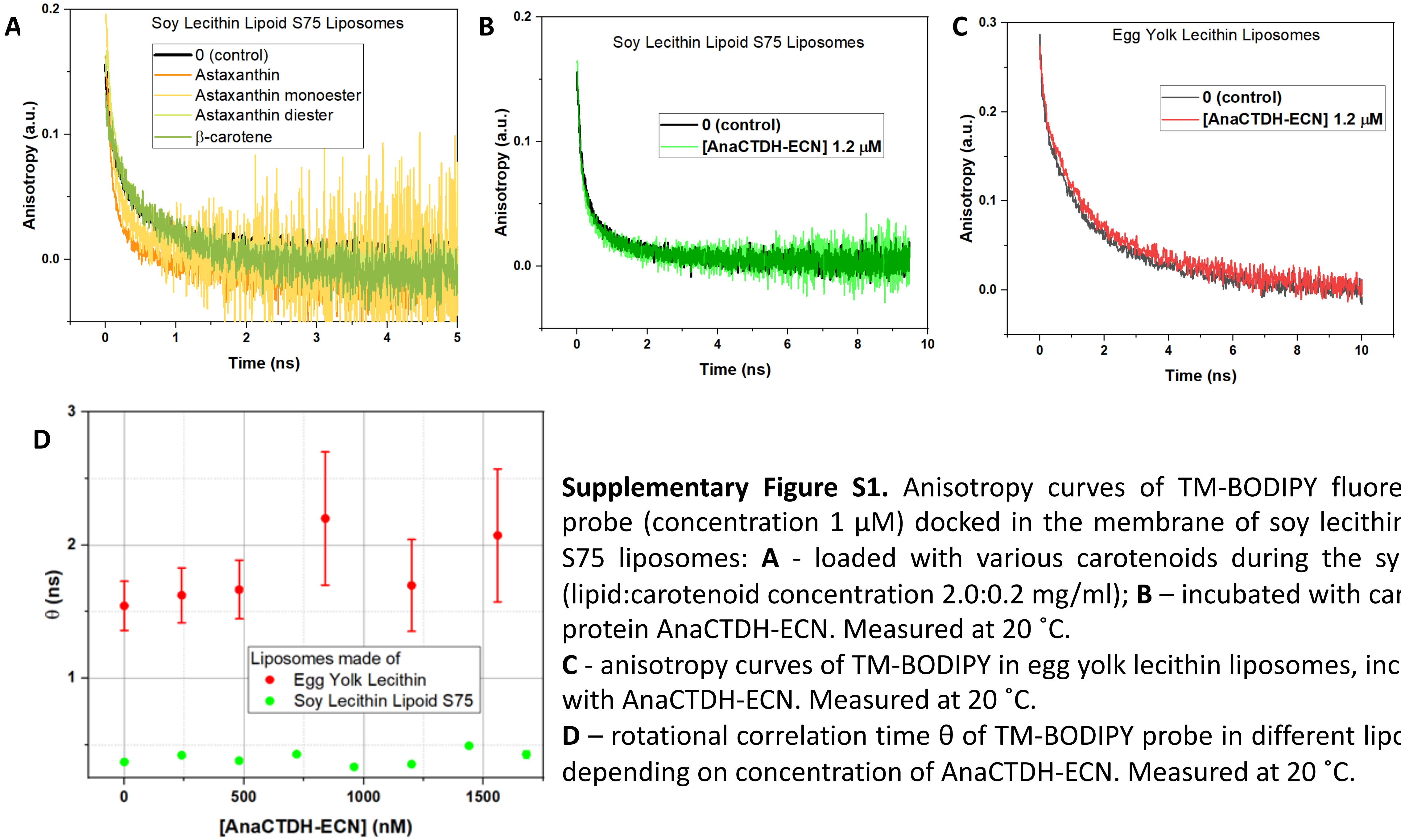

Supplementary Figure S1. Anisotropy curves of TM-BODIPY fluorescence probe (concentration 1 μM) docked in the membrane of soy lecithin lipoid S75 liposomes: **A** - loaded with various carotenoids during the synthesis (lipid:carotenoid concentration 2.0:0.2 mg/ml); **B** – incubated with carotenoprotein AnaCTDH-ECN. Measured at 20 ˚C.
C - anisotropy curves of TM-BODIPY in egg yolk lecithin liposomes, incubated with AnaCTDH-ECN. Measured at 20 ˚C.
D – rotational correlation time θ of TM-BODIPY probe in different liposomes depending on concentration of AnaCTDH-ECN. Measured at 20 ˚C.

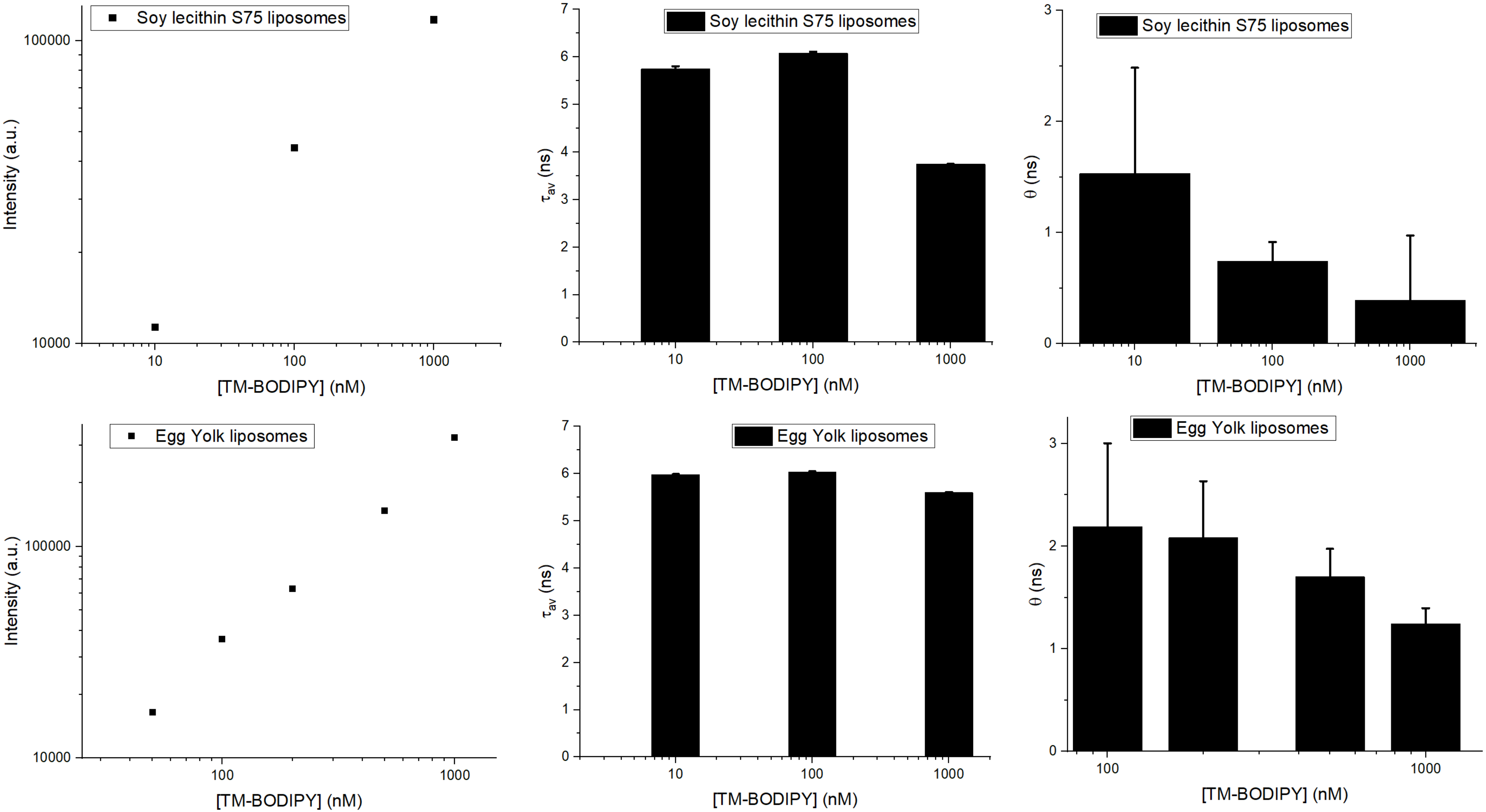

Supplementary Figure S2. Dependence of total intensity, mean lifetime and correlation rotation time on concentration of TM-BODIPY probe in different liposomes (role of homoFRET effects).

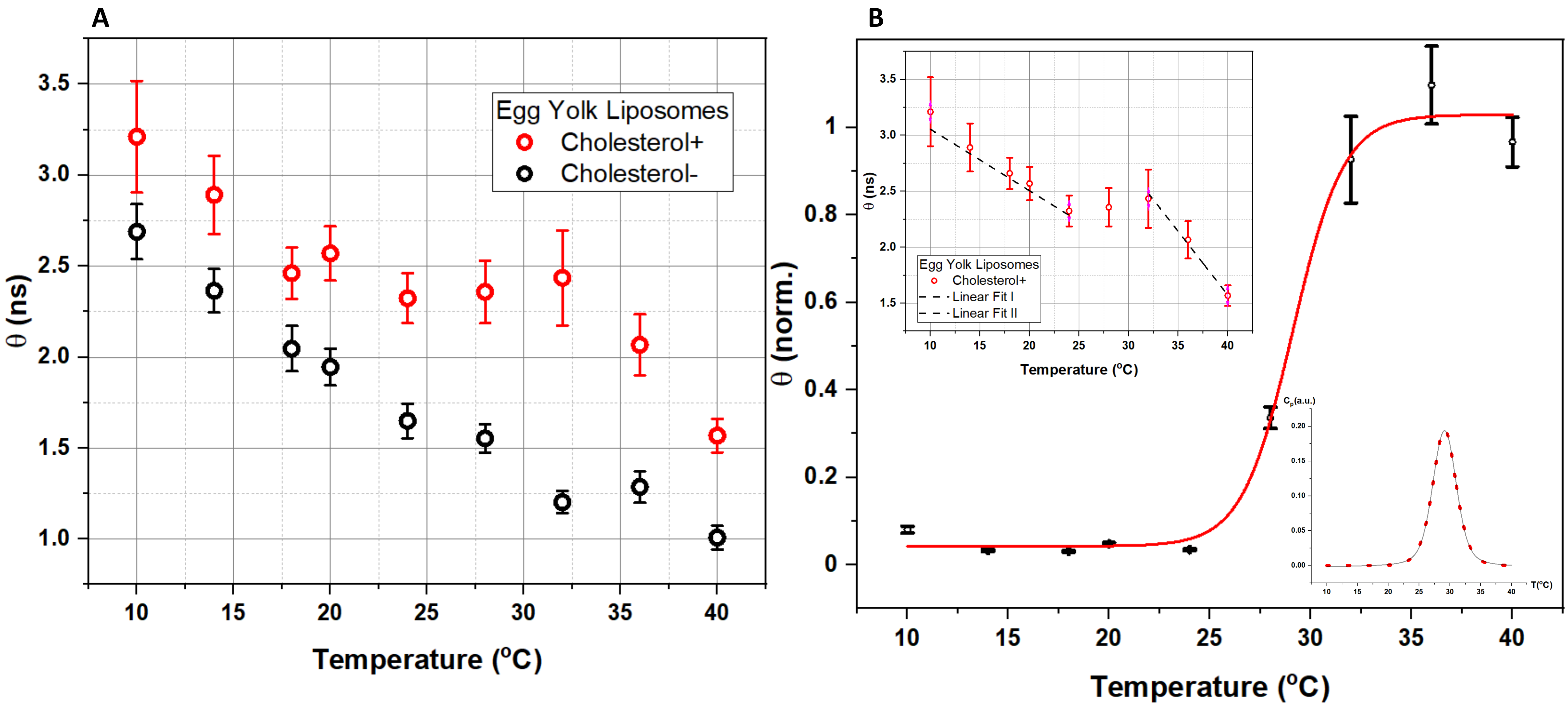

Supplementary Figure S3. Effect of cholesterol, loaded into raw egg yolk liposomes, on membrane microviscosity (**A**) and occurrence of phase transition (**B**).

Supplementary Table S1. Parameters of the phospholipid membrane that was used in molecular dynamics computational stimulations.

POPC		120
POPE		32
PLPE		16
PLPC		20
SLPC		30
SLPE		12
PC	PhosphatidylCholine	
PE	PhosphatidylEtanolamine	
S	Stearic acid	18:0
P	Palmitoleic acid	16:1(9)
O	Oleic acid	18:1(9)
L	Linoleic acid	18:2(9,12)

Supplementary Table S2. Size distribution and polydispersity index (PDI) of studied liposomes.

Type of Liposomes	Carotenoid concentration (mg/mL)	Diameter (nm)	%	PDI
S75 (intact)	-	118±56	96.6	0.269
S75:β-Car	0.008	126±62	96.6	0.349
S75:β-Car	0.040	116±33	84	0.348
S75:β-Car	0.200	403±121	70.8	0.511
S75:Asta	0.002	96±42	99	0.260
S75:Asta	0.020	121±96	95.4	0.401
S75:Asta	0.250	155±98	96.9	0.451
S75:Asta-Esters	0.250	270±182	90	0.341
S75:Asta-Esters	0.500	290±180	99	0.262
S75:Asta-Esters	0.800	300±253	93	0.398
Egg Yolk Lecithin Liposomes (intact)	-	100±10	99	0.520
Egg Yolk Lecithin Liposomes: cholesterol	-	148±34	99	0.500
